# Association of the total cholesterol–high-density lipoprotein–glucose index with metabolic-associated steatotic liver disease: a 5-year retrospective cohort study

**DOI:** 10.3389/fmed.2026.1782215

**Published:** 2026-03-20

**Authors:** Jun Liu, Jie Liu, Jiaqian Zhu, Chuang Gao, Xun Jiang

**Affiliations:** 1 Department of Emergency, Shenzhen New Frontier United Family Hospital, Shenzhen, Guangdong, China; 2 Department of Neurology, Yiwu Central Hospital, Yiwu, Zhejiang, China; 3 School of Medicine, The First Affiliated Hospital of Shenzhen University, Shenzhen Second Peoples Hospital, Shenzhen University, Shenzhen, Guangdong, China; 4 Department of Emergency, Shenzhen Dapeng New District Kuichong People’s Hospital, Shenzhen Guangdong, China

**Keywords:** dyslipidemia, glucose metabolism, metabolic dysfunction-associated steatotic liver disease, nonlinearity, total cholesterol, high-density lipoprotein, and glucose index

## Abstract

**Objective:**

Current research on the relationship between the Total Cholesterol, High-Density Lipoprotein, and Glucose (CHG) index and the risk of Metabolic-Associated Steatotic Liver Disease (MASLD) is still limited. This study aims to explore the relationship between them and their predictive value in Chinese adults.

**Methods:**

A retrospective cohort analysis enrolled 6,274 individuals who received health assessments at Shenzhen Kuichong People’s Hospital from 2018 to 2023. Cox proportional hazards regression analysis was implemented to evaluate associations between CHG and MASLD risk. Cox proportional hazards regression model with restricted cubic spline functions was utilized to assess potential non-linear associations. Additionally, ROC analysis evaluated CHG’s predictive ability for MASLD.

**Results:**

Multivariable Cox regression showed that for each 0.1 unit increase in CHG, the HR for MASLD risk was 1.055 (95% CI: 1.030, 1.080). Non-linear association between them was identified, with inflection point at CHG = 5.42. Left of this inflection point, HR for CHG (per 0.1-unit)-MASLD association was 1.088 (95% CI: 1.055–1.122); right of this inflection point, the HR was 0.969 (95% CI: 0.912–1.029). Additionally, ROC curve analysis demonstrated that the AUC of CHG for predicting the 5-year risk of MASLD reached 0.678, which exceeded those of any single component indicator. When compared with TyG and FLI, CHG exhibited comparable predictive performance with no statistically significant differences. Time-dependent ROC analysis showed that within the 2.0–5-year period, the AUC of CHG remained between 0.656 and 0.678.

**Conclusion:**

This study indicates that increased CHG is independently positively associated with the risk of MASLD and exhibits a non-linear association. Reducing CHG values below 5.42 and implementing further reduction measures may significantly decrease the risk of MASLD. CHG exhibits certain predictive value for MASLD risk and has the potential to serve as an early identification marker for high-risk populations, providing a novel perspective for clinical prevention strategies of MASLD.

## Introduction

Metabolic dysfunction-Associated Steatotic Liver Disease (MASLD) is defined as a condition characterized by hepatic steatosis accompanied by one or more cardiometabolic risk factors, including hypertension (HTN), hyperglycemia, obesity, and dyslipidemia (DLP) (1). Recent comprehensive meta-analyses demonstrate that the global prevalence of MASLD reaches 30%, representing a 50% increase from 1990 to 2019, with Southeast Asia and South Asia showing particularly high prevalence rates of 33, and 44%, respectively (2). Notably, hepatic steatosis represents the key pathological feature of MASLD, and the disease can progress from benign simple steatosis to severe pathological changes including inflammation, fibrosis, and even malignant transformation (3, 4). Furthermore, MASLD is frequently associated with various extrahepatic metabolic disorders, including elevated blood pressure, elevated uric acid, DLP, elevated blood glucose, and insulin resistance (IR), which may lead to serious complications such as atherosclerotic cardiovascular disease (ASCVD), diabetes mellitus (DM), and other manifestations of metabolic syndrome (5–8). Among them, ASCVD is the leading cause of death and disability worldwide, and increasing evidence indicates that MASLD can independently further aggravate this disease burden by significantly increasing the risk of ischemic heart disease, ischemic stroke, and peripheral artery disease (9, 10). Therefore, identifying predictive factors for MASLD is crucial for disease prevention and management, as this facilitates screening of high-risk populations and implementation of intervention measures, effectively delaying or preventing disease progression and ultimately improving patient outcomes.

Studies have shown that reduced high-density lipoprotein cholesterol (HDL-c) levels lead to impaired cholesterol efflux capacity and diminished antioxidant properties, both of which may contribute to the pathogenesis of MASLD (1). Moreover, HDL-c levels have been confirmed to be significantly inversely associated with MASLD risk (11). Additionally, the total cholesterol/HDL (TC/HDL-c) ratio is also associated with increased MASLD risk (12). Similarly, impaired glucose metabolism is closely linked to MASLD risk (13). Evidence suggests that hyperglycemia promotes hepatic lipid accumulation through activation of *de novo* lipogenesis, induction of oxidative stress, and inflammatory responses, thereby triggering the onset and progression of MASLD (13–15). Furthermore, elevated Fasting Plasma Glucose (FPG) levels have been demonstrated to be positive relationship with MASLD incidence (16). Therefore, compared with traditional single indicators, composite indices integrating lipid and glucose parameters may better capture the complex metabolic characteristics of MASLD and are expected to provide more comprehensive information for MASLD risk assessment.

In recent years, Amin Mansoori et al. proposed a novel indicator termed the Total Cholesterol, High-density lipoprotein, and Glucose (CHG) index, which comprises TC, HDL-c, and FPG (17). This index has demonstrated significant clinical value in predicting diabetic microvascular complications and assessing risks of cardiovascular events, HTN, stroke, and cardiometabolic diseases (18–21). We speculate that a positive association may exist between CHG and MASLD. Regrettably, limited research has addressed this topic. One study confirmed a positive association between CHG levels and the risk of fatty liver; however, that study employed conventional ultrasonographic criteria for fatty liver diagnosis, rather than the MASLD-based definition, which additionally requires the concurrent presence of at least one cardiometabolic risk factor (22). Therefore, we conducted a retrospective cohort study aimed at exploring the relationship between the CHG index and MASLD risk, as well as further assessing the predictive value of the CHG for MASLD.

## Materials and methods

### Study design and study population

This investigation adopted a retrospective cohort framework. Study participants comprised individuals who received standardized health evaluations at Kuichong People’s Hospital in Dapeng New District, Shenzhen, from January 2018 through December 2023. The CHG index served as the independent variable, while MASLD was the dependent variable (employing binary coding: 0 representing non-MASLD, 1 representing MASLD).

**FIGURE 1:**
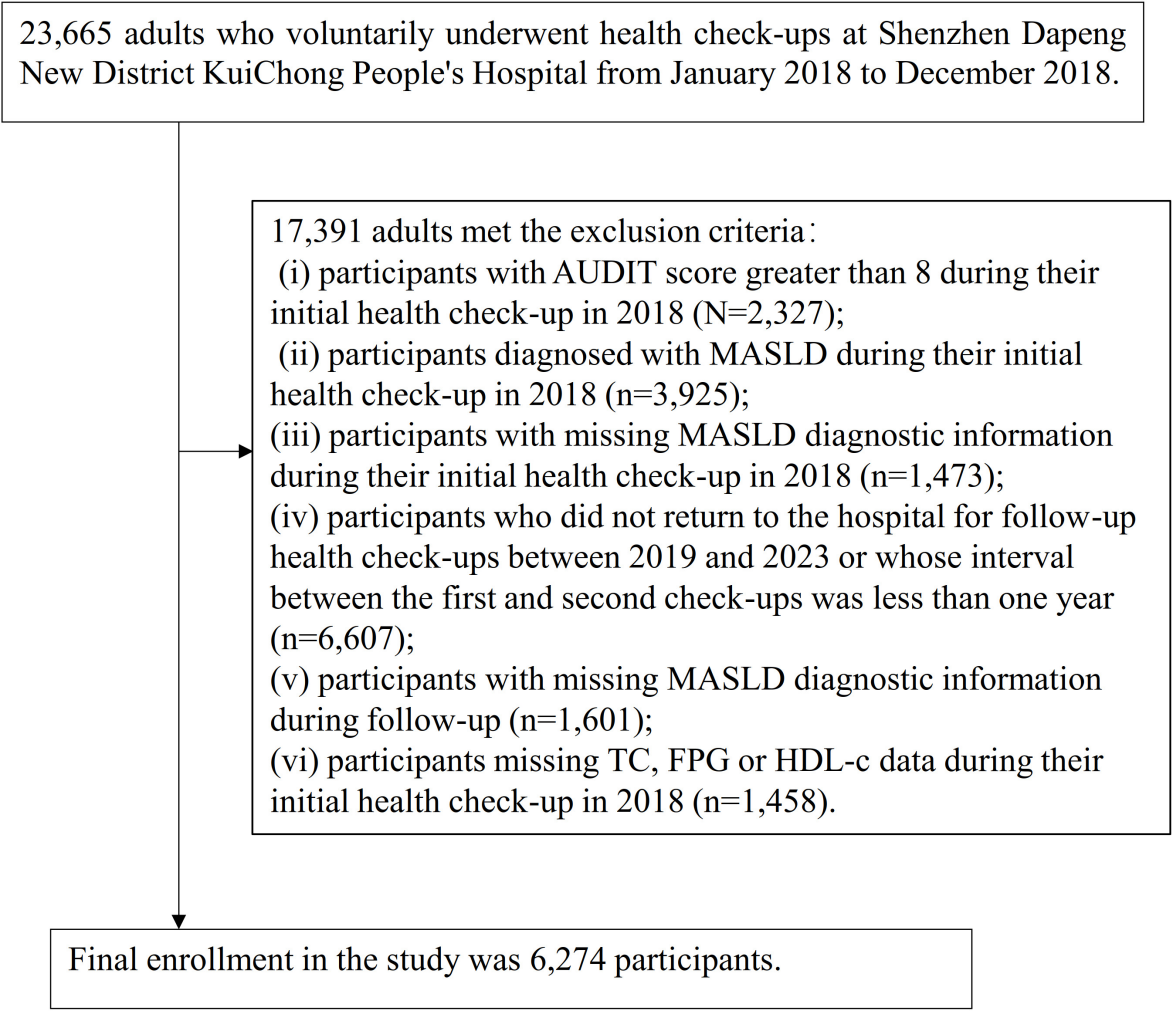
Flow diagram depicting participant inclusion and exclusion.

The initial study population included 23,665 participants aged 18 years and above who completed routine physical examinations between January and December 2018. Among them, 17,391 participants were excluded based on specific criteria: (i) those with an Alcohol Use Disorders Identification Test (AUDIT) score exceeding 8 points at the first examination in 2018 (*n* = 2,327) (23); (ii) those diagnosed with MASLD at the first examination in 2018 (*n* = 3,925); (iii) participants lacking MASLD diagnostic information at the first examination in 2018 (*n* = 1,473); (iv) individuals who failed to reappear for clinical assessments throughout the 2019–2023 period, or whose duration between the initial and subsequent visit was under 12 months (*n* = 6,607); (v) participants with insufficient diagnostic data for MASLD during follow-up (*n* = 1,601); (vi) those with incomplete baseline records in 2018 regarding HDL-c, FPG, or TC (*n* = 1,458). Consequently, the ultimate study population comprised 6,274 participants. The comprehensive selection sequence is depicted in Figure 1.

### Ethical approval and consent

Formal ethics authorization was granted by the Institutional Review Board (IRB) associated with Kuichong People’s Hospital, located in Shenzhen’s Dapeng New District (Approval No. 2024005). Due to the retrospective design and complete de-identification of all participant data, informed consent requirements were waived by the IRB. The research was conducted in strict accordance with the principles outlined in the Declaration of Helsinki and adhered to all applicable ethical guidelines and requirements (24).

### Variables

#### The total cholesterol, high-density lipoprotein, and glucose index

The CHG index was evaluated as a continuous variable. This index was calculated according to the following formula: CHG = Ln [TC (mg/dL)*FPG (mg/dL)/2*HDL-c (mg/dL)], where “n” denotes the natural logarithm with base e (17).

#### Assessment of MASLD and follow-up

At each follow-up visit, participants underwent liver ultrasonography (US) examination to evaluate the degree of fatty liver. Each US examination was conducted by at least two certified US specialists to enhance diagnostic accuracy and minimize observer bias. Specifically, one sonographer was responsible for conducting the examination and making the initial interpretation, while a senior physician reviewed the findings. This two-step review process-comprising an initial examiner and a reviewing physician—ensured that every US conclusion was scrutinized for consistency and accuracy. The examination was performed using Mindray US equipment (DC-35Pro), and participants were required to fast for at least 6 h prior to the examination. Certified US specialists interpreted the US images with knowledge of the participants’ clinical and laboratory data. The diagnosis of fatty liver was based on enhanced liver echogenicity (brightness) compared to the right kidney parenchyma. In conjunction with the US examination, individuals completed a standardized AUDIT questionnaire comprising 10 scoring items (0–4 points each). The AUDIT is a standardized alcohol risk assessment tool that has been extensively validated internationally, and its threshold of < 8 is widely recognized as an effective screening criterion for low-risk alcohol consumption, which is consistent with the core requirement of “low/no alcohol intake” as defined in the 2023 Delphi consensus (23). Therefore, in this study, the diagnosis of MASLD required both (1) an AUDIT score < 8 and (2) confirmation of hepatic steatosis by US examination (25). Additionally, at least one of the following cardiometabolic risk factors had to be present: HTN (systolic blood pressure, SBP ≥ 140 mmHg or diastolic blood pressure, DBP ≥ 90 mmHg), overweight or obesity (body mass index, BMI ≥ 25 kg/m^2^), glucose metabolism abnormalities (FPG ≥ 5.6 mmol/L or history of DM), and DLP (defined as meeting any of the following criteria: TG ≥ 1.70 mmol/L; TC ≥ 5.2 mmol/L; LDL-c ≥ 3.4 mmol/L; HDL-c < 1.04 mmol/L; fulfillment of any single criterion was considered as DLP) (26–28).

For participants diagnosed with MASLD during subsequent follow-up examinations, the follow-up duration was calculated as the interval between the first MASLD diagnosis and the baseline assessment. For participants not diagnosed with MASLD, the follow-up duration was the interval between the last follow-up examination and the baseline assessment.

### Covariates

Covariate selection was based on our clinical expertise and insights from existing literature (17, 22). Included covariates were: (i) Continuous variables: Hemoglobin A1c (HBA1c), age, LDL-c, Waist Circumference (WC), Aspartate Aminotransferase (AST), Height (HT), High-Sensitivity C-Reactive Protein (HS-CRP), BMI, DBP, Serum Creatinine (Scr), Gamma-Glutamyl Transferase (GGT), TG, Weight (WT), SBP, Alanine Aminotransferase (ALT); (ii) Categorical variables: sex, DM, current smoking, DLP, HTN, antihypertensive medication (HTN-MED), antihyperlipidemic medication (DLP-MED), antidiabetic medication (DM-MED) and physical activity level.

### Data collection and definitions

Comprehensive physical assessments were conducted by professionally trained clinical staff using a standardized questionnaire for participants undergoing health examinations. The assessment covered sociodemographic characteristics (such as sex, age), behavioral factors (such as smoking), medical history (such as HTN, DM, and medication status), and other important information. Alcohol consumption was assessed using the AUDIT, and physical activity was assessed using the International Physical Activity Questionnaire Short Form (IPAQ-SF) (25). Blood pressure measurements were taken using a standard mercury sphygmomanometer. Participants were required to fast for at least 10 h before each examination, after which venous blood samples were collected. Blood samples were analyzed for TC, TG, HDL-c, LDL-c, and FPG using a Beckman 5800 fully automated analyzer.

The definitions and calculations of BMI, Triglyceride-Glucose (TyG) Index, and Fatty Liver Index (FLI) were as follows: BMI was calculated as body weight (kg) divided by height squared (m^2^). TyG index was defined as ln [FPG (mg/dL)*TG (mg/dL)/2] (29). FLI was calculated using the logistic function: FLI = e^∧^X/(1 + e^∧^X) × 100, where X = 0.953* ln TG (mg/dL) + 0.139 *BMI + 0.718*ln GGT (U/L) + 0.053*WC (cm) − 15.745, with “Ln” denoting the natural logarithm (base e) (30).

### Handling of missing data

Missing data is a common and unavoidable situation in observational studies. In this analysis, multiple variables had missing values, including smoking status (302, 4.81%), WC (35, 0.56%), SBP (27, 0.43%), DBP (27, 0.43%), DLP (121, 1.93%), Scr (139, 2.22%), AST (123, 1.96%), ALT (134, 2.14%), and GGT (85, 1.35%). To minimize the potential bias caused by missing data, we employed multiple imputations by chained equations (MICE) to handle missing values (31, 32). This approach is based on the chained equations framework, with algorithm-specific imputation strategies applied to variables with missing data: continuous variables were imputed using Predictive Mean Matching (PMM), and categorical variables were imputed using logistic regression imputation, conducted over 10 iterations. Variables included in the imputation model were: age, SBP, DBP, WT, HT, BMI, WC, LDL-c, TG, HbA1c, AST, ALT, Scr, HS-CRP, GGT, DM, HTN, DLP, HTN-MED, DLP-MED, DM-MED, current smoking, physical activity, and sex. The outcome variable (incident MASLD) was not included in the imputation model for baseline covariates, consistent with the temporal logic of the cohort study design. According to established analytical criteria, missing values were assumed to be missing at random (MAR) (32). It should be noted that five imputed datasets were generated, allowing each dataset to be analyzed independently for estimates such as means and regression coefficients. The resulting estimates were subsequently pooled using Rubin’s rules.

### Statistical analysis

Participants were stratified by CHG quartiles to compare their baseline characteristics. Data for continuous metrics with a normal distribution were reported using mean ± standard deviation (SD), whereas those lacking normality were summarized as median and interquartile range (IQR). Categorical parameters were expressed as counts alongside their respective percentages. Comparisons of continuous variables among groups were conducted using analysis of variance (ANOVA) or the Kruskal-Wallis test, while categorical variables were compared using the chi-square test (χ^2^).

This study used Cox proportional hazards regression models to investigate the association between CHG and MASLD risk. Three models were constructed: (i) Model I: adjusted for sex and age; (ii) Model II: adjusted for sex, age and BMI; (iii) Fully adjusted Model III: adjusted for age, sex, BMI, GGT, SBP, TG, ALT, HS-CRP, HbA1c, current smoking, physical activity, HTN, DM, DM-MED, DLP-MED, HTN-MED. Multicollinearity among covariates was assessed using the Variance Inflation Factor (VIF), with a VIF value > 5 considered as the threshold for detecting multicollinearity. WC exhibited VIF values exceeding this threshold and were therefore excluded from the multivariate models (Supplementary Table 1).

Previous studies have confirmed significant associations between HTN, obesity, and glucose metabolism with MASLD (13, 33, 34). To verify the robustness of our findings, sensitivity analyses were conducted by excluding participants with BMI ≥ 28 kg/m^2^ (35), and participants diagnosed with DM and HTN, respectively. Second, since multicollinearity was shown to exist between WC and BMI, and **WC** was therefore excluded from the multivariable model, and given that WC may be considered to better reflect metabolic risk in Asian populations than BMI, an additional sensitivity analysis was conducted in which WC was substituted for BMI as the adjustment variable in the multivariable Cox regression model to verify the stability of the findings (36). Moreover, to further investigate whether the association between CHG and MASLD is solely dependent on strict alcohol consumption restriction, a sensitivity analysis was performed based on the metabolic dysfunction-associated alcohol-related liver disease (MetALD) definition. MetALD is characterized by the presence of hepatic steatosis and at least one cardiometabolic risk factor, while higher levels of alcohol consumption are allowed (37). Furthermore, considering potential bias that might be introduced during multiple imputation of missing covariates, a complete case sensitivity analysis was conducted by re-evaluating the association between CHG and MASLD after participants with missing values in any covariate were excluded. Additionally, given that a substantial proportion of participants (*n* = 6,607) were excluded due to loss to follow-up or failure to return for subsequent assessments, which could potentially affect the association between CHG and MASLD, the baseline characteristics in 2018 between participants included in the final analysis and those who were excluded were compared to assess whether selection bias existed. Finally, the E-value was calculated to examine the possible effect of unmeasured and unknown confounding variables on the association between CHG and MASLD risk (38). Furthermore, to ensure that the multiple imputation process did not alter the distribution characteristics of the original data and did not introduce additional bias, the baseline characteristics of variables with missing values before and after imputation were compared.

Subgroup analyses were conducted using stratified Cox regression models, with stratification based on age, sex, BMI, AST, ALT, GGT, SBP, DBP, current smoking, DM-MED, DLP, and physical activity. According to clinical thresholds, the following variables were categorized: age was divided into five groups (< 30 years, 30–40 years, 40–50 years, 50–60 years, and ≥ 60 years); SBP was classified into two groups based on a cutoff of 140 mmHg (< 140 mmHg and ≥ 140 mmHg); DBP was classified into two groups based on a cutoff of 90 mmHg (< 90 mmHg and ≥ 90 mmHg) (39); AST and ALT were each divided into two groups based on a cutoff of 40 U/L (< 40 U/L and ≥ 40 U/L) (40); GGT levels were categorized by sex-specific thresholds: males were divided into two groups based on a cutoff of 60 U/L (low: ≤ 60 U/L vs. high: > 60 U/L), while females were divided using a cutoff of 40 U/L (low: ≤ 40 U/L vs. high: > 40 U/L) (40). BMI was classified into four groups according to the Chinese adult BMI classification criteria: underweight (< 18.5 kg/m^2^), normal weight (18.5–23.9 kg/m^2^), overweight (24.0–27.9 kg/m^2^), and obesity (≥ 28.0 kg/m^2^) (41). The adjusted model contained variables including age, sex, BMI, GGT, SBP, TG, ALT, HS-CRP, HbA1c, current smoking, physical activity, HTN, DM, DM-MED, DLP-MED, HTN-MED, while excluding stratification variables. Log-likelihood ratio tests were used to compare models with and without interaction terms to evaluate potential interactions. The subgroup-specific HRs with 95% CIs and the corresponding *P-*values for interaction were presented in the form of a forest plot.

Developed a Cox proportional hazards regression model using restricted cubic spline functions to explore potential non-linear relationships between CHG and MASLD risk. Upon detecting non-linear relationships, a recursive algorithm was employed to identify the inflection point. Subsequently, separate Cox regression models were developed on either side of the inflection point. Log-likelihood ratio tests were employed to determine the most appropriate model for describing the association between CHG and MASLD risk. Finally, to verify the robustness of the observed non-linear relationship between CHG and MASLD, sensitivity analyses were conducted by separately excluding participants using DLP-Med and DM-Med.

Finally, ROC curves were constructed to assess the predictive ability of TC, FPG, HDL-c, TyG, FLI, and CHG in predicting the risk of MASLD within 5 years. Additionally, time-dependent ROC analysis was performed to evaluate the predictive performance of CHG at different time points during follow-up, specifically at 2.0, 3.0, 4.0, and 5.0 years (42). Simultaneously, the corresponding AUC, best threshold, sensitivity, and specificity were calculated.

The findings were reported in compliance with the STROBE statement guidelines (43). Statistical analyses were conducted utilizing R software (version 3.4.3) and Empower software (version 4.2). A two-tailed *P-*value of less than 0.05 was deemed statistically significant.

## Results

### Study population characteristics

**FIGURE 2:**
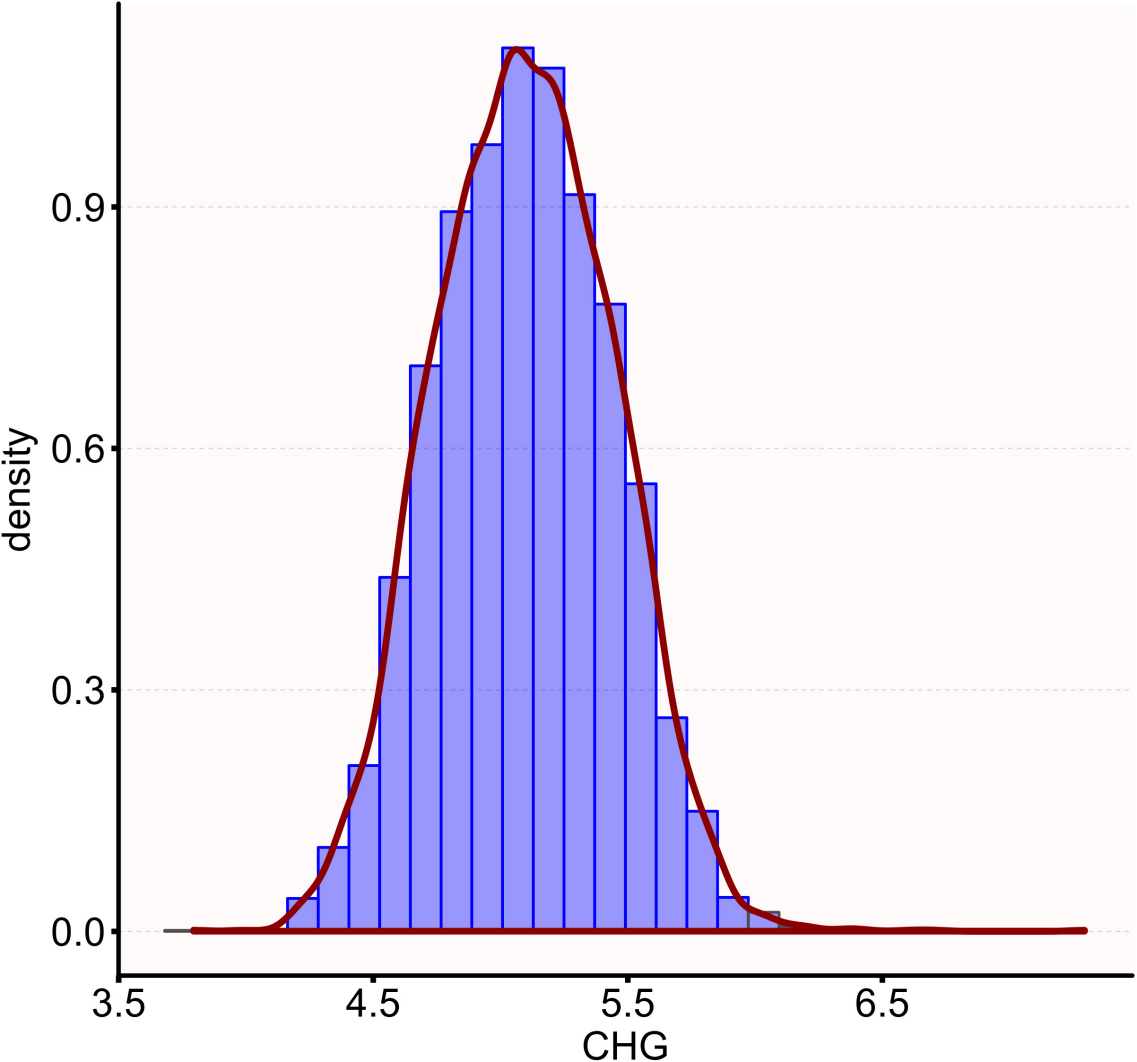
Distribution of CHG. The distribution appeared normal distribution, spanning from 3.79 to 7.29, with a mean (SD) value of 5.09 (0.34).

Table 1 presents the demographic and clinical characteristics of 6,274 participants, of whom males accounted for 67.42% of the total population. As shown in Figure 2, the CHG distribution was normally distributed, ranging from 3.79 to 7.29, with a mean ± SD value of 5.09 ± 0.34. Participants were divided into four groups according to CHG quartiles: Q1 group (< 4.85), Q2 group (4.85–5.09), Q3 group (5.09–5.34), and Q4 group (≥ 5.34). Compared with the Q1 group, participants in the higher quartile groups had higher SBP, AST, DBP, WC, FLI, TyG, LDL-c, BMI, TG, HbA1c, FPG, ALT, age, TC, GGT levels, and relatively lower HDL-c levels. Compared with the Q1 group, the higher quartile groups had a higher proportion of males and higher proportions of those with HTN, DLP, HTN-MED, DLP-MED, and low physical activity.

**TABLE 1:** Baseline characteristics according to CHG quartiles.

CHG quartiles	Q1 (< 4.85)	Q2 (4.85–5.09)	Q3 (5.09–5.34)	Q4 (≥ 5.34)	*P*-value
Participants	1,569	1,568	1,568	1,569	
Age, years	39.05 ± 8.12	40.83 ± 8.96	41.70 ± 8.53	42.11 ± 8.25	< 0.001
SBP, mmHg	109.16 ± 10.91	113.88 ± 11.27	116.37 ± 10.63	118.97 ± 11.48	< 0.001
DBP, mmHg	71.12 ± 7.41	74.00 ± 7.47	75.68 ± 7.08	77.28 ± 7.42	< 0.001
BMI, kg/m^2^	23.25 ± 2.89	24.58 ± 2.95	25.37 ± 2.89	26.26 ± 2.84	< 0.001
WC, cm	80.27 ± 9.81	85.79 ± 10.14	89.91 ± 8.59	93.27 ± 8.42	< 0.001
TC, mg/dL	173.94 ± 28.96	185.50 ± 29.33	197.31 ± 30.13	220.87 ± 35.54	< 0.001
LDL-c, mg/dL	91.88 ± 21.69	113.25 ± 22.31	128.46 ± 25.07	149.47 ± 32.81	< 0.001
HDL-c, mg/dL	66.00 ± 12.97	53.95 ± 9.13	46.88 ± 7.61	39.44 ± 7.07	< 0.001
TG, mg/dL	80.30 ± 33.29	91.56 ± 34.43	110.50 ± 42.99	161.70 ± 111.38	< 0.001
HbA1c, %	4.42 ± 0.23	4.57 ± 0.24	4.67 ± 0.27	4.79 ± 0.44	< 0.001
FPG, mg/dL	80.05 ± 6.69	84.32 ± 6.96	87.21 ± 7.64	90.58 ± 12.72	< 0.001
AST, U/L	25.67 ± 12.53	27.37 ± 8.72	28.74 ± 12.80	29.27 ± 8.88	< 0.001
ALT, U/L	28.89 ± 13.26	33.47 ± 14.33	37.24 ± 16.23	40.94 ± 18.11	< 0.001
Scr, mmol/L	71.46 ± 15.44	70.72 ± 13.73	69.11 ± 14.93	70.89 ± 15.42	0.676
HS-CRP, mg/L	0.90 (0.40–2.30)	0.90 (0.49–2.00)	1.00 (0.50–2.00)	1.10 (0.60–2.30)	0.566
GGT, U/L	18.00 (14.00–25.00)	22.00 (17.00–30.00)	25.00 (19.00–34.00)	30.00 (23.00–41.00)	< 0.001
FLI	10.45 (5.67–21.01)	21.05 (11.25–35.60)	33.09 (20.03–48.96)	51.28 (34.57–67.82)	< 0.001
TyG	8.00 ± 0.38	8.19 ± 0.35	8.41 ± 0.35	8.79 ± 0.43	< 0.001
DM, n%	5 (0.32%)	3 (0.19%)	11 (0.70%)	22 (1.40%)	< 0.001
HTN, n%	55 (3.51%)	105 (6.70%)	116 (7.40%)	136 (8.67%)	< 0.001
DLP, n%	171 (10.90%)	317 (20.22%)	397 (25.32%)	581 (37.03%)	< 0.001
HTN-MED, n%	61 (3.89%)	109 (6.95%)	120 (7.65%)	126 (8.03%)	< 0.001
DLP-MED, n%	90 (5.74%)	139 (8.86%)	150 (9.57%)	101 (6.44%)	< 0.001
DM-MED, n%	18 (1.15%)	19 (1.21%)	22 (1.40%)	26 (1.66%)	0.605
Current smoking, n%	99 (6.31%)	92 (5.87%)	135 (8.61%)	146 (9.31%)	< 0.001
Physical activity, n%					
Sedentary	280 (17.85%)	270 (17.22%)	274 (17.47%)	330 (21.03%)	< 0.001
Low	556 (35.44%)	602 (38.39%)	578 (36.86%)	646 (41.17%)	
Moderate	556 (35.44%)	511 (32.59%)	562 (35.84%)	491 (31.29%)	
High	177 (11.28%)	185 (11.80%)	154 (9.82%)	102 (6.50%)	
Sex					
Female	1,092 (69.60%)	595 (37.95%)	250 (15.94%)	107 (6.82%)	< 0.001
Male	477 (30.40%)	973 (62.05%)	1,318 (84.06%)	1,462 (93.18%)	

Continuous variables were summarized as mean (SD) or medians (quartile interval); categorical variables were displayed as percentage (%): SBP, systolic blood pressure; HDL-c, High-Density Lipoprotein Cholesterol; CHG, total cholesterol, high-density lipoprotein, and glucose; AST, Aspartate Aminotransferase; LDL-c, Low-Density Lipoprotein Cholesterol; FPG, Fasting Plasma Glucose; BMI, Body Mass Index; TG, Triglycerides; WC, Waist Circumference; TC, Total Cholesterol; ALT, Alanine Aminotransferase; HS-CRP, High-Sensitivity C-Reactive Protein; DBP, diastolic blood pressure; HbA1c, Hemoglobin A1c; Scr, Serum Creatinine; GGT, Gamma-Glutamyl Transferase; DM, Diabetes Mellitus; DLP, Dyslipidemia; HTN, Hypertension; HTN-MED, antihypertensive medication; DLP-MED, antihyperlipidemic medication; TyG, Triglyceride-Glucose Index; FLI, Fatty Liver Index; DM-MED, antidiabetic medication.

### Incidence of MASLD

During a median follow-up period of 2.72 years, 1,104 incident MASLD cases (17.6%) were identified among 6,274 participants. After stratification by CHG quartiles, the MASLD incidence rates (per 1,000 person-years) were: Q1 27.93, Q2 61.35, Q3 84.06, Q4 131.26. The overall cumulative MASLD incidence was 17.6%, with specific cumulative incidence rates for each quartile group as follows: Q1 6.50%, Q2 14.03%, Q3 19.26%, Q4 30.59%. Notably, compared with the Q1 group (lowest CHG level), the Q4 group (highest CHG level) had a significantly higher MASLD incidence (P for trend < 0.001) (Table 2).

**TABLE 2:** Incidence rate of MASLD (% or per 1,000 person-year).

CHG quartiles	Participants (n)	MASLD events (n)	Incidence rate (95% CI),%	Per 1,000 person-year
Total	6,274	1,104	17.60 (16.65–18.55)	76.24
Q1 (< 4.85)	1,569	102	6.50 (5.28–7.72)	27.93
Q2 (4.85–5.09)	1,568	220	14.03 (12.31–15.75)	61.35
Q3 (5.09–5.34)	1,568	302	19.26 (17.31–21.21)	84.06
Q4 (≥ 5.34)	1,569	480	30.59 (28.30–32.88)	131.26

CI, confidence; n, number; MASLD, Metabolic dysfunction-Associated Steatotic Liver Disease.

**FIGURE 3:**
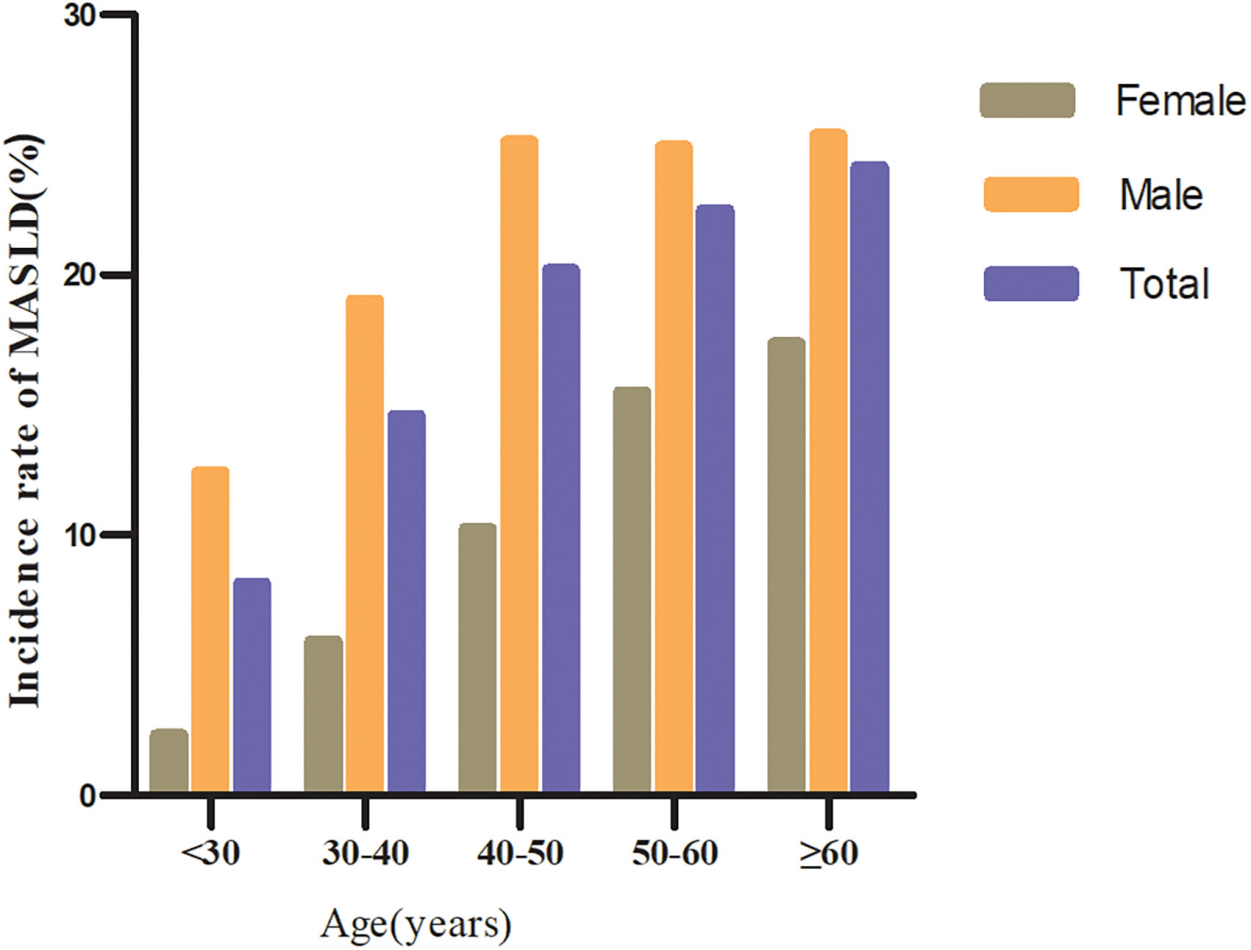
Incidence of MASLD stratified by age (in 10-year intervals) and sex.

Age was divided into five groups: < 30 years, 30–40 years, 40–50 years, 50–60 years, and ≥ 60 years. Across all age groups, males had a higher incidence of MASLD than females (Figure 3). Furthermore, regardless of sex, MASLD incidence increased with age.

### The relationship between CHG and the risk of MASLD.

To investigate the association between CHG and MASLD risk, three Cox proportional hazards regression models were developed. In Model I, for every 0.1-unit increase in CHG, the risk of MASLD increased by 15.6% (HR = 1.156, 95% CI: 1.137–1.176). Model II revealed that each 0.1-unit increment in CHG was linked to an 8% increase in MASLD risk (HR = 1.080, 95% CI: 1.059–1.102). In the full adjusted Model III, a 5.5% increase in MASLD risk was observed for each 0.1-unit increase in CHG (HR = 1.055, 95% CI: 1.030–1.080) (Table 3).

**TABLE 3:** The relationship between CHG and the risk of MASLD.

Exposure	Model I (HR, 95%CI) P	Model II (HR, 95%CI) P	Model III (HR, 95%CI) P
CHG (per 0.1-unit)	1.156 (1.137, 1.176) < 0.001	1.080 (1.059, 1.102) < 0.001	1.055 (1.030, 1.080) < 0.001
CHG quartiles			
Q1	Ref	Ref	Ref
Q2	2.203 (1.742, 2.786) < 0.001	1.577 (1.241, 2.005) < 0.001	1.506 (1.184, 1.917) < 0.001
Q3	3.025 (2.417, 3.786) < 0.001	1.760 (1.387, 2.232) < 0.001	1.602 (1.258, 2.039) < 0.001
Q4	4.710 (3.804, 5.832) < 0.001	2.297 (1.817, 2.903) < 0.001	1.878 (1.467, 2.403) < 0.001
P for trend	< 0.001	<0.001	< 0.001

CHG, total cholesterol, high-density lipoprotein, and glucose; CI, confidence interval, Ref, reference; HR, Hazard ratios. Model I: we adjusted sex, age. Model II: we adjusted sex, age, BMI. Model III: we adjusted age, sex, BMI, GGT, SBP, TG, ALT, HS-CRP, HbA1c, current smoking, physical activity, HTN, DM, DM-MED, DLP-MED, HTN-MED.

Furthermore, after stratification by CHG quartiles, the data were re-entered into the Cox regression model. Using Q1 as the reference group, multivariable-adjusted analysis showed: HR of 1.506 (95% CI: 1.184–1.917) for Q2, 1.602 (95% CI: 1.258–2.039) for Q3, and 1.878 (95% CI: 1.467–2.403) for Q4. These results indicate that compared with Q1, the MASLD risk increased by 50.6, 60.2, and 87.8% for Q2, Q3, and Q4, respectively (Table 3, Model III).

### Sensitivity analysis

Several sensitivity evaluations were conducted to ensure the robustness of our findings. Initially, after omitting individuals with DM and correcting for potential confounders, a 5.3% rise in MASLD risk was observed for every 0.1-unit increment in CHG (HR = 1.053, 95% CI: 1.028–1.079). This relationship persisted when the cohort was limited to participants with BMI < 28 kg/m^2^, yielding an HR of 1.063 (95% CI: 1.033–1.094). Finally, among participants without HTN, the association remained statistically significant with an HR of 1.054 (95% CI: 1.026–1.079) (Table 4). Furthermore, when WC was substituted for BMI as the adjustment variable in the multivariable Cox regression model, CHG (per 0.1-unit increase) was found to be associated with an HR of 1.064 (95% CI: 1.042–1.088) for MASLD, which was highly consistent with the main analysis (Supplementary Table 2, Model I). Moreover, in the complete case sensitivity analysis, all participants with missing values in any covariate were excluded, and the analysis was re-evaluated. The results showed that CHG (per 0.1-unit increase) was associated with an HR of 1.074 (95% CI: 1.050–1.098) for MASLD, which was generally consistent with the results from the multiple imputation analysis (Supplementary Table 2, Model II).

**TABLE 4:** The relationship between CHG and the risk of MASLD in different sensitivity analysis.

Exposure	Model I (HR, 95%CI) *P*-value	Model II (HR, 95%CI) *P*-value	Model III (HR, 95%CI) *P*-value
CHG (per 0.1-unit)	1.053 (1.028, 1.079) < 0.001	1.063 (1.033, 1.094) < 0.001	1.054 (1.026, 1.079) < 0.001
CHG quartiles			
Q1	Ref	Ref	Ref
Q2	1.501 (1.179, 1.910) 0.00096	1.465 (1.117, 1.922) 0.00584	1.569 (1.218, 2.021) < 0.001
Q3	1.589 (1.247, 2.025) 0.00018	1.680 (1.280, 2.205) 0.00019	1.648 (1.278, 2.124) < 0.001
Q4	1.856 (1.448, 2.379) < 0.00001	2.013 (1.520, 2.666) < 0.00001	1.857 (1.431, 2.411) < 0.001
P for trend	< 0.001	<0.001	< 0.001

Model I involved sensitivity analyses after excluding participants with DM (N = 6,233). Age, sex, BMI, GGT, SBP, TG, ALT, HS-CRP, HbA1c, current smoking, physical activity, HTN, DM-MED, DLP-MED, and HTN-MED were adjusted. Model II involved a sensitivity analysis of participants with BMI < 28 kg/m^2^ (*n* = 5,401). Age, sex, BMI, GGT, SBP, TG, ALT, HS-CRP, HbA1c, current smoking, physical activity, HTN, DM, DM-MED, DLP-MED, HTN-MED were adjusted. Model III involved sensitivity analyses after excluding participants with HTN (*N* = 5,862). age, sex, BMI, GGT, SBP, TG, ALT, HS-CRP, HbA1c, current smoking, physical activity, DM, DM-MED, DLP-MED, HTN-MED were adjusted. CHG, total cholesterol, high-density lipoprotein, and glucose; CI, confidence interval, Ref, reference; HR, Hazard ratios.

Additionally, the relationship between CHG and MetALD was further explored, and a positive association was demonstrated, with an HR of 1.086 (95% CI: 1.063–1.110) (Supplementary Table 2, Model III). The baseline characteristics in 2018 between participants included in the final analysis and those who were excluded were further compared, and the results revealed no significant differences in nearly all baseline characteristics between the two groups (Supplementary Table 3). Furthermore, baseline characteristics of variables with missing values before and after imputation were compared, and no significant differences were observed between the two datasets (Supplementary Table 4).

Additionally, the E-value was 1.24, which was higher than the relative risk of the association between CHG and potential unmeasured confounders (1.22), but lower than the relative risk of the association between unmeasured confounders and MASLD (1.25). This suggests that unknown or unmeasured confounders were unlikely to significantly affect the association between CHG and MASLD risk. These sensitivity analyses strengthened the credibility and robustness of our findings.

### Subgroup analysis

In the prespecified subgroup analyses, no significant interactions between CHG and age, sex, BMI, AST, ALT, GGT, SBP, DBP, current smoking, DM-MED, DLP, or physical activity were observed (all P for interaction > 0.05). These results indicate that the aforementioned factors did not significantly influence or modify the association between CHG and MASLD risk. The subgroup-specific HRs (95% CIs) and the corresponding P values for interaction are detailed in the forest plot (Supplementary Figure 1 and Supplementary Table 5).

### Non-linear relationship between CHG and the risk of MASLD.

**FIGURE 4:**
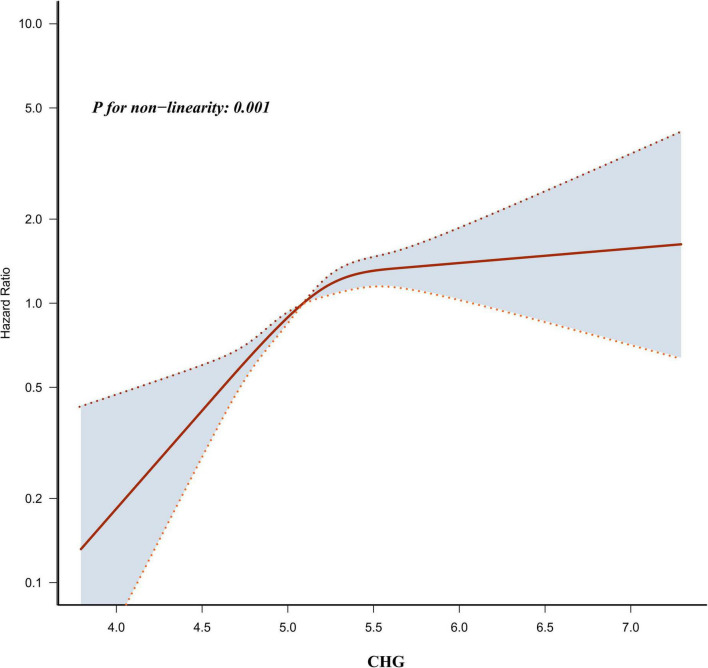
Non-linear relationship between CHG and the risk of MASLD.

By integrating restricted cubic splines into the Cox regression analysis, a non-linear association between CHG and MASLD risk was observed (P for non-linearity < 0.05; Figure 4). The inflection point for CHG was determined to be 5.42 using a recursive algorithm. A two-piecewise Cox regression model was applied to estimate the HRs and CIs on either side of the inflection point. Below the 5.42, every 0.1-unit increment in CHG was tied to an 8.8% elevation in MASLD risk (HR = 1.088, 95% CI: 1.055–1.122). Conversely, above the inflection point, each 0.1-unit increase in CHG was associated with an HR of 0.969 (95% CI: 0.912–1.029) for MASLD risk, which did not reach statistical significance (Table 5).

**TABLE 5:** The result of two-piecewise linear regression model.

Outcome: Incident MASLD	HR (95%CI)	*P*-value
Inflection points of CHG	5.42	< 0.001
<5.42 (per 0.1-unit)	1.088 (1.055, 1.122)	
≥5.42 (per 0.1-unit)	0.969 (0.912, 1.029)	0.300
P for log-likelihood ratio test	0.001	

Adjusted age, sex, BMI, GGT, SBP, TG, ALT, HS-CRP, HbA1c, current smoking, physical activity, HTN, DM, DM-MED, DLP-MED, HTN-MED. CHG, total cholesterol, high-density lipoprotein, and glucose; CI, confidence interval, Ref, reference; HR, Hazard ratios; MASLD, Metabolic Dysfunction-Associated Steatotic Liver Disease.

To assess the robustness of the observed non-linear relationship, sensitivity analyses were performed separately excluding participants using DLP-MED and DM-MED. The results showed that below the inflection point of 5.42, for each 0.1-unit increase in CHG, MASLD risk increased by 9.7% (excluding DLP-MED: HR = 1.097, 95% CI: 1.061–1.135) and by 10.8% (excluding DM-MED: HR = 1.088, 95% CI: 1.054–1.123, *p* < 0.001) respectively. Above the inflection point, the association between CHG and MASLD was not statistically significant in either analysis (excluding DLP-MED: HR = 0.982, 95% CI: 0.923–1.044; excluding DM-MED: HR = 0.965, 95% CI: 0.907–1.026). These findings demonstrate that the saturation effect above the inflection point persists after excluding users of the aforementioned medications, with the direction and magnitude of HRs consistent with the primary analysis (Supplementary Table 6).

### ROC analysis of the predictive value of CHG, FPG, TC, HDL-c, TyG, and FLI for MASLD

**FIGURE 5:**
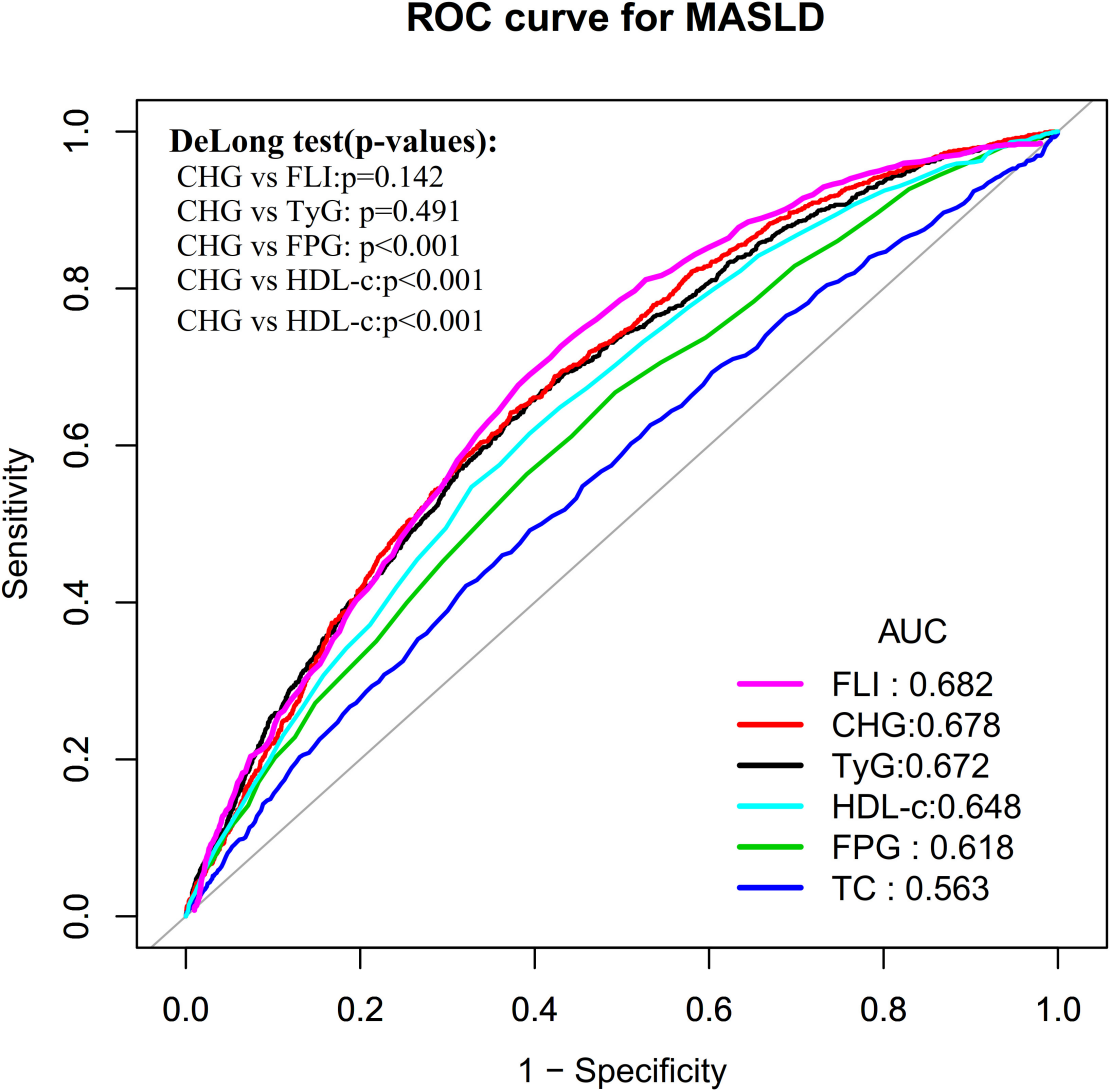
ROC curves for CHG, FPG, TC, HDL-c, TyG, and FLI prediction of MASLD.

ROC curves were constructed to evaluate the predictive capability of CHG, FPG, TC, HDL-c, TyG and FLI for MASLD risk (Figure 5). The AUC values for each variable were ranked as follows: FLI: 0.682 > CHG: 0.678 > TyG > 0.672 > HDL-c: 0.648 > FPG: 0.618 > TC: 0.563. The Youden index values for CHG, FPG, TC, HDL-c, FLI, and TyG were 0.270, 0.175, 0.100, 0.221, 0.275, and 0.262, respectively, with corresponding best threshold values of 5.17, 84.50, 206.50, 47.50, 30.65, and 8.39 (Table 6). These results indicated that both the AUC and Youden index of CHG were superior to those of its individual constituent components (FPG, TC, and HDL-c), suggesting that CHG provides modest incremental predictive value over single components. Compared with the TyG index and FLI, CHG demonstrated comparable predictive performance with no statistically significant differences (DeLong test, *P* > 0.05).

**TABLE 6:** ROC analysis of the predictive value of CHG, FPG, TC, HDL-c TyG, and FLI for MASLD.

Test	AUC(95%CI)	Best threshold	Specificity	Sensitivity	Youden index
CHG	0.678(0.661–0.694)	5.17	0.627	0.642	0.270
TyG	0.672(0.655–0.690)	8.39	0.604	0.658	0.262
FPG (mg/dL)	0.618(0.600–0.636)	84.50	0.508	0.668	0.175
TC (mg/dL)	0.563(0.544–0.582)	206.50	0.679	0.421	0.100
HDL-c (mg/dL)	0.648(0.630–0.665)	47.50	0.606	0.615	0.221
FLI	0.682(0.673–0.691)	30.65	0.596	0.679	0.275

AUC, area under the curve; CI, Confidence interval; TC, Total Cholesterol; FPG, Fasting Plasma Glucose; CHG, total cholesterol, high-density lipoprotein, and glucose; TyG, Triglyceride-Glucose Index; FLI, Fatty Liver Index; HDL-c, High-Density Lipoprotein Cholesterol.

### Time-dependent ROC analysis of the predictive value of CHG for MASLD risk

To further evaluate the predictive capability of CHG for MASLD risk across different time periods, time-dependent ROC analysis was performed. The results demonstrated that the AUC values for predicting MASLD incidence at 2.0, 3.0, 4.0, and 5 years were 0.656 (95% CI: 0.635–0.677), 0.666 (95% CI: 0.647–0.683), 0.669 (95% CI: 0.651–0.687), and 0.678 (95% CI: 0.662–0.695), respectively. These findings indicate that CHG possessed relatively stable predictive value for MASLD risk across both short- and long-term follow-up periods (Supplementary Figures 2 and Supplementary Table 7).

## Discussion

This study demonstrates that CHG is independently and positively associated with MASLD risk. Furthermore, a saturation effect curve was observed with an inflection point at a CHG value of 5.42, exhibiting different association patterns on either side of this threshold. ROC curve analysis revealed that CHG provides modest incremental predictive value for 5-year MASLD incidence compared with its individual constituent components. Time-dependent ROC analysis demonstrates that the predictive value of CHG for MASLD risk remained relatively stable during the follow-up period of 2.0–5 years.

Accumulating evidence demonstrates that any form of DLP—whether occurring independently or in combination with other lipid abnormalities—is closely associated with increased MASLD risk (44–46). Based on a cross-sectional investigation of 4,498 healthy individuals, a decreased risk of MASLD was linked with elevated HDL-c (OR 0.074; 95% CI: 0.044–0.123) (47). Conversely, higher TC levels indicated a positive relationship with increased MASLD prevalence (OR 1.242; 95% CI: 1.122–1.374) (47). Additionally, another cohort study involving 32,121 participants showed a significant dose-response relationship between the TC/HDL-c ratio and MASLD risk after adjusting for multiple confounding factors (12). Similarly, glucose metabolism abnormalities are closely related to MASLD risk (13). A cross-sectional study of 42,091 young adults showed that elevated FPG (mmol/L) were positively relationship with MASLD risk (OR = 1.69, 95% CI: 1.58–1.80) (16). Furthermore, a prospective cohort study involving 2,467 participants conducted a multivariable Cox regression analysis, which indicated that compared to the lowest quartile of fasting glucose variation coefficient (CV-FG), the highest quartile had a HR of 2.80 (95% CI: 1.69–4.64) for non-alcoholic fatty liver disease (NAFLD) (48). The CHG index was first proposed by Mansoori et al., integrating TC, HDL-c, and FPG into a novel composite metabolic indicator; initial validation demonstrated its superior discriminatory ability for T2DM diagnosis compared with its individual components (17). Subsequently, the clinical application of CHG has progressively expanded to a broader spectrum of cardiometabolic conditions. Mo et al. conducted a large-scale cohort study directly comparing CHG and TyG in predicting CVD risk, finding that the two indices demonstrated comparable overall predictive performance, yet each exhibited distinct advantages in specific metabolic subgroups—CHG performed better in populations with prominent cholesterol metabolic dysregulation, whereas TyG was more predictive in those with predominant triglyceride-driven IR—suggesting that the two indices reflect different yet complementary pathophysiological pathways (18). Zeng et al., using data from the CHARLS cohort, confirmed that higher CHG levels were independently associated with increased stroke risk (19). Tian et al. demonstrated through longitudinal analysis that higher cumulative CHG burden was independently associated with elevated risks of both CVD incidence and all-cause mortality, underscoring that sustained metabolic dysregulation reflected by CHG—rather than a single measurement—confers progressively greater cardiometabolic risk (21). Furthermore, a study based on data from 13,286 MASLD adults in the NHANES cohort (1999–2018) found that the CHG index was also significantly and positively associated with both all-cause mortality and cardiovascular mortality in MASLD patients, with HRs of 1.50 (95% CI: 1.30, 1.74) and 1.53 (95% CI: 1.16, 2.02), respectively (49). The associations between CHG, as a reliable composite metabolic index, and a wide range of cardiometabolic diseases and outcomes have thus been validated. Therefore, we hypothesize that the composite index CHG, which involves TC, TG, and HDL-c, may be positively relationship with MASLD risk. Unfortunately, research on this topic remains scarce, with one study having examined the association between the CHG index and fatty liver disease risk (22). This study indicated that for each 1-unit increase in CHG, the risk of fatty liver increased by 1.46 times (HR = 2.46, 95% CI: 1.68–3.58) (22). The present study confirmed our hypothesis: elevated CHG levels are independently and positively associated with MASLD risk. In addition, to minimize information loss and more accurately quantify the association between CHG and outcomes, we analyzed CHG as both categorical and continuous variables. In addition, sensitivity analyses were conducted separately in participants with BMI below 28 kg/m^2^, without HTN, and without DM. Furthermore, analyses were conducted across different analytical frameworks to assess the association between CHG and MASLD: WC was substituted for BMI as the adjustment variable in the fully adjusted model, analysis was performed based on the MetALD definition with relaxed alcohol consumption criteria, and complete case analysis was conducted. The results confirmed that these findings were consistent in these specific subgroups.

It is worth noting that the present study employed abdominal US as the reference standard for hepatic steatosis. Compared with the gold standard for hepatic fat quantification, MRI proton density fat fraction (MRI-PDFF) has significantly superior detection sensitivity for mild steatosis compared with abdominal US (50). Therefore, in this study, some participants in the early stage of mild steatosis were classified as non-MASLD, thereby leading to underestimation of the incidence of outcome events, and at the same time possibly causing the observed HR to undergo attenuation toward the null. Furthermore, the CHG index integrates markers reflecting lipid metabolic dysregulation and glucose metabolic abnormalities, disturbances that tend to emerge prior to hepatic fat accumulation reaching the ultrasonographically detectable threshold (generally requiring steatosis exceeding 20%). Consequently, the metabolic sensitivity of CHG may precede the morphological detection threshold of ultrasonography, resulting in a certain “temporal window misalignment” between them. This implies that the association observed in the present study between CHG and MASLD incidence more accurately reflects its predictive capacity for moderate-to-severe MASLD. However, even in the presence of these two conditions that tend to bias the results toward null findings between CHG and MASLD, our analysis still identified an independent association between them. In summary, identifying CHG as a risk factor for MASLD and elucidating its association has important clinical significance. Incorporating CHG into routine clinical assessment may help clinicians optimize risk stratification and management strategies. Increasing regular physical activity and improving dietary patterns (such as reducing TC and FPG levels) may help reduce the incidence of MASLD.

In addition, the study by Li et al. also evaluated the predictive ability of the CHG index for fatty liver (22). Their study reported an AUC of 0.645 for CHG in predicting the risk of fatty liver, whereas the present study found an AUC of 0.678 for CHG in predicting MASLD incidence within 5 years (22). The possible explanations for this discrepancy may include differences in follow-up duration, outcome variable definitions, and sex distribution, among others. Notably, several alternative composite metabolic indices for assessing hepatic steatosis have been proposed in recent years, including the TyG index and FLI, among others (51, 52). ROC curve analysis in the present study demonstrated that the predictive performance of CHG for MASLD was non-inferior to that of TyG and FLI. Additionally, CHG exhibited incremental predictive value over its individual component indices. The TyG index, as a composite of TG and FPG, primarily captures TG metabolic dysregulation and IR (53). Previous studies have confirmed that elevated TC can induce endoplasmic reticulum stress and mitochondrial dysfunction through free cholesterol lipotoxicity, thereby promoting the onset and progression of MASLD (54). In contrast, CHG captures a composite metabolic disturbance signal reflecting the interaction between cholesterol metabolic imbalance and hyperglycemia, representing a mechanistic complement rather than an overlap with—TyG. CHG is essentially a logarithmic integration of the TC/HDL-c ratio and FPG, reflecting the synergistic pathogenic effects of cholesterol metabolic imbalance and glucotoxicity. Accordingly, TyG and CHG target distinct metabolic axes and are mechanistically complementary. In particular, CHG may provide additional early risk warning signals in the early stages of metabolic dysregulation when TG levels have not yet risen significantly. Moreover, time-dependent ROC analysis revealed that the AUC values of CHG remained stable between 0.656 and 0.678 over a prediction horizon of 2.0–5.0 years, indicating relatively consistent predictive performance for both short- and long-term MASLD incidence. Therefore, as a novel, clinically accessible, and reproducible composite index, CHG holds promise as an early marker for identifying individuals at high risk of MASLD, providing a valuable reference for future MASLD risk prediction models. Future research may explore integrating CHG with traditional risk factors and emerging biomarkers (e.g., Atherogenic Index of Plasma, TyG, and HOMA-IR) within a machine learning framework to develop more concise and efficient risk prediction models, thereby further enhancing the capacity for early screening of high-risk MASLD populations (29, 55).

The mechanisms linking elevated CHG levels to the risk of MASLD remain unclear, but they are likely related to abnormalities in lipid and glucose metabolism. Research indicates that hyperglycemia activates *de novo* lipogenesis in the liver and induces oxidative stress and inflammation, promoting lipid accumulation in hepatocytes and contributing to MASLD progression (13–15). Additionally, low HDL-c levels impair cholesterol efflux and reduce antioxidant and anti-inflammatory effects, further increasing oxidative stress and injury in the liver (1). Studies have also shown a significant negative association between HDL-c levels and MASLD risk (47). Elevated TC promotes the development and progression of MASLD through free cholesterol toxicity-induced endoplasmic reticulum stress and mitochondrial dysfunction (54). Consequently, the CHG index, which integrates TC, HDL-c, and FPG, reflects the dual dysmetabolism of cholesterol metabolism imbalance and glucose toxicity, thereby providing a more comprehensive and quantitative characterization of the metabolic features underlying MASLD.

A Cox proportional hazards regression model with a restricted cubic spline function was employed, revealing a non-linear association between CHG and the risk of MASLD, with an inflection point identified at 5.42. When CHG is less than 5.42, each increase of 0.1 units in CHG is associated with an 8.8% rise in MASLD risk. However, when CHG exceeds 5.42, this association is no longer statistically significant. Notably, we also conducted sensitivity analyses by separately excluding participants using DLP-MED and DM-MED to reassess the relationship between CHG and MASLD. The results indicated that the non-linear relationship persisted after excluding users of these two categories of medications, with the direction and magnitude of HRs on both sides of the inflection point in these two subgroups remaining consistent with those of the primary analysis. In other words, higher levels of CHG are associated with an increased risk of MASLD, but this effect stabilizes once CHG surpasses the critical value of 5.42. The possible reasons for the emergence of this phenomenon are as follows: First, analysis indicated that participants with CHG ≥ 5.42 had higher levels of age, SBP, DBP, BMI, WC, LDL-c, TG, HbA1c, AST, ALT, HS-CRP, and GGT compared to those with CHG < 5.42. In addition, participants with CHG ≥ 5.42 also had higher proportions of DM, HTN, DLP, HTN-MED, DM-MED, current smoking, sedentary lifestyle, and low physical activity (Supplementary Table 8). These factors are closely related to the risk of MASLD (3, 16, 47, 56). In the cohort with CHG < 5.42, the levels of these risk factors were lower, which may enhance the impact of CHG on MASLD risk. Conversely, when CHG ≥ 5.42, the presence of these risk factors may weaken the impact of CHG on MASLD risk. Second, this study employed abdominal US as the reference standard for MASLD diagnosis. However, US has well-known saturation limitations in detecting hepatic steatosis—particularly under conditions of high hepatic lipid burden, its detection sensitivity is compromised (57). When the baseline hepatic lipid burden in the high-CHG population has already approached the upper detection limit of ultrasonography, the absolute increment in newly diagnosed MASLD rate during the 5-year follow-up period is consequently constrained, thus presenting statistically as a plateau effect in the relative risk increase. Furthermore, under conditions of severe metabolic derangement, the liver may, through activating lipid export-related pathways (such as increased VLDL secretion) or upregulation of fatty acid β-oxidation and other compensatory mechanisms, partially alleviate the net hepatic lipid accumulation rate, such that further elevated CHG fails to bring proportional increases in MASLD risk, thereby forming a biologically authentic non-linear threshold effect (58). Recognizing this non-linear pattern holds considerable clinical utility, providing guidance for clinical consultation and optimization of MASLD prevention strategies. Specifically, through dietary interventions and lifestyle modifications—ensuring CHG remains under the 5.42 threshold and achieves further declines—could substantially mitigate the incidence of MASLD

This study has the following important advantages: (i) In this cohort study, by analyzing the relationship between CHG and MASLD using both continuous and categorical variables, information loss was minimized, thereby enabling a more comprehensive and accurate assessment of the association between them. This study also explores non-linear relationships, marking a significant advancement. (ii) ROC curve analysis indicates that CHG has certain predictive value for MASLD risk. (iii) The use of multiple imputations to handle missing data significantly enhances statistical efficiency and reduces bias due to missing covariate information. (iv) To validate the robustness of the results, multiple sensitivity analyses were conducted: categorizing CHG as a categorical variable; assessing potential biases due to unobserved and unknown confounding factors using E-values; and re-evaluating the association after excluding participants with obesity (BMI ≥ 28 kg/m^2^), HTN, or DM. Subgroup analyses demonstrated that age, sex, SBP, DBP, current smoking, DM-MED, DLP, or physical activity did not significantly modify the association between CHG and MASLD risk.

However, several limitations of this study should be acknowledged. First, the study population was restricted to Chinese participants, which limits the external validity and generalizability of the findings to other racial/ethnic groups or geographic regions; validation in multicenter, multiregional, and ethnically diverse populations is therefore needed. Second, the diagnosis of MASLD in this study was based on routine ultrasound (US). Although US is widely used in clinical practice and large-scale health screening due to its non-invasive, convenient, and cost-effective nature, its sensitivity for diagnosing MASLD remains limited. We plan to conduct prospective cohort studies employing more objective diagnostic techniques, such as MRI-PDFF, to more accurately assess MASLD and validate our findings. Third, the relatively high loss-to-follow-up rate in the present study may have some impact on the stability of the results. To assess potential selection bias, we compared baseline characteristics between participants included in the final analysis and those who were excluded; the results demonstrated that baseline characteristics were largely balanced between the two groups, suggesting a low risk of selection bias. Future studies should further reduce the loss-to-follow-up rate by establishing well-structured follow-up management systems, thereby enhancing the reliability of the findings. Fourth, given the retrospective cohort study design, the present study has limited capacity to adjust for potential confounding factors such as dietary habits, chronic hepatitis B, autoimmune liver disease, and insulin levels. Nevertheless, the calculated E-values suggested that these unknown and unmeasured confounders are unlikely to exert a substantial impact on our conclusions. Future prospective cohort studies incorporating a broader range of confounding factors will be conducted to further validate these findings. Fifth, in the present study, an AUDIT score of < 8 was employed as a screening tool for low-risk alcohol consumption, which differs to some extent from the alcohol gram-based threshold defined in the 2023 Delphi consensus. Future prospective studies should therefore incorporate detailed information on absolute alcohol intake in grams to address this limitation. Sixth, considering the potential competing risk between all-cause mortality and MASLD outcomes, competing risk analysis could not be performed in this study due to its retrospective nature and the absence of mortality registry data. However, as this study is a health examination cohort comprising individuals with relatively good overall health status, the all-cause mortality rate during follow-up was low. When the incidence of competing events is low, the Fine-Gray model and the Cox model tend to yield consistent results with negligible differences; the Cox model therefore remains reasonably applicable in this context, and the conclusions are considered relatively reliable (59). Future efforts will focus on linking the database with death registration systems to systematically evaluate the potential impact of competing risks on the study findings. Finally, although this cohort analysis established an independent association between CHG and the incidence of MASLD, a causal relationship cannot be determined from the current evidence alone.

## Conclusion

This study found an independent positive and non-linear relationship between CHG and the risk of MASLD, with a turning point at a CHG value of 5.42. ROC curve analysis indicated that, compared with its individual components, CHG has certain incremental predictive value for 5-year MASLD incidence. Time-dependent ROC analysis demonstrated that the predictive value of CHG for MASLD risk remained relatively stable during the follow-up period from 2.0 to 5 years. As a simple and easily obtainable composite indicator, CHG is expected to become a marker for identifying high-risk populations for MASLD, providing new insights for optimizing clinical prevention and management of MASLD.

## Data Availability

The raw data supporting the conclusions of this article will be made available by the author, without undue reservation.
