# Supplementary material for: Association of the total cholesterol–high-density lipoprotein–glucose index with metabolic-associated steatotic liver disease: a 5-year retrospective cohort study
Source: Front Med (Lausanne). 2026 Mar 20;13:1782215. doi: 10.3389/fmed.2026.1782215 (PMC13047192; doi:10.3389/fmed.2026.1782215)
Supplement: Supplementary file 1 [file Data_Sheet_1.docx]

**Association of the Total Cholesterol–High-Density Lipoprotein–Glucose (CHG) index with Metabolic-Associated Steatotic Liver Disease: a 5-year retrospective cohort study**

**Running title: CHG and MASLD**

**Jun Liu****^1^, Jie Liu^1^, Jiaqian Zhu^2,3^ Chuang Gao^4^, Xun Jiang^1#^**

^1^Department of Emergency, Shenzhen New Frontier United Family Hospital, Shenzhen, 518038, Guangdong Province, China.

^2^Department of Neurology, Yiwu Central Hospital, 699 Jiangdong Road, Yiwu, 322000, Zhejiang, China.

^3^School of Medicine, Shenzhen University, The First Affiliated Hospital of Shenzhen University, Shenzhen Second People's Hospital, Shenzhen 518000, Guangdong Province, China.

^4^Department of Emergency, Shenzhen Dapeng New District Kuichong People's Hospital, Shenzhen 518000, Guangdong Province, China.

**Corresponding author**

**Xun Jiang**

Department of Emergency, Shenzhen New Frontier United Family Hospital

**No. 4012 Fuqiang Road, Futian District,**

Shenzhen, 518038,

Guangdong Province,

China.

Email: [373166605@qq.com](mailto:373166605@qq.com)

**Table S1. collinearity screening**

|  | Step 1 | Step 2 | Step 3 | Step 4 | Step 5 |
| --- | --- | --- | --- | --- | --- |
| CHG | 38.8 | 38.8 | 29.2 | 4.4 | 4.4 |
| age, years | 1.3 | 1.3 | 1.3 | 1.3 | 1.3 |
| DM, n% | 1.6 | 1.6 | 1.6 | 1.6 | 1.6 |
| HTN, n% | 3.6 | 3.6 | 3.6 | 3.6 | 3.6 |
| DLP, n% | 1.3 | 1.3 | 1.3 | 1.3 | 1.3 |
| HTN-MED, n% | 3.6 | 3.6 | 3.6 | 3.6 | 3.6 |
| DLP-MED, n% | 1.4 | 1.4 | 1.4 | 1.4 | 1.4 |
| DM-MED, n% | 1.6 | 1.6 | 1.6 | 1.6 | 1.6 |
| SBP, mmHg | 3 | 3 | 3 | 3 | 3 |
| DBP, mmHg | 2.9 | 2.9 | 2.9 | 2.9 | 2.9 |
| BMI, kg/m² | 4.4 | 4.4 | 4.4 | 4.4 | 3.1 |
| WC, cm | 4.7 | 4.7 | 4.7 | 4.7 | NA |
| Current smoking, n% | 1 | 1 | 1 | 1 | 1 |
| LDL-c, mg/dL | 39.7 | 39.7 | 8.2 | 2.4 | 2.4 |
| TC, mg/dL | 5 | 5 | 2.2 | 1.5 | 1.5 |
| HbA1c, % | 4.5 | 4.5 | 3.8 | 1.7 | 1.7 |
| AST, U/L | 1.7 | 1.7 | 1.7 | 1.7 | 1.7 |
| ALT, U/L | 2.2 | 2.2 | 2.2 | 2.2 | 2.2 |
| HS-CRP, mg/L | 1 | 1 | 1 | 1 | 1 |
| GGT, U/L | 1.5 | 1.5 | 1.4 | 1.4 | 1.4 |
| Physical activity, n% | 1.1 | 1.1 | 1.1 | 1.1 | 1.1 |
| Sex | 2.8 | 2.8 | 2.8 | 2.7 | 2.1 |

Variables excluded from collinearity screening: WC

Abbreviations: SBP, systolic blood pressure; DBP, diastolic blood pressure; HDL-c, High-Density Lipoprotein Cholesterol; CHG，total cholesterol, high-density lipoprotein, and glucose; DM, Diabetes Mellitus; DLP, Dyslipidemia; HTN, Hypertension; HTN-MED, antihypertensive medication; DLP-MED, antihyperlipidemic medication; DM-MED, antidiabetic medication; AST, Aspartate Aminotransferase; LDL-c, Low-Density Lipoprotein Cholesterol; GGT, Gamma-Glutamyl Transferase; BMI, Body Mass Index; WC, Waist Circumference; TC, Total Cholesterol; HbA1c, Hemoglobin A1c; HS-CRP, High-Sensitivity C-Reactive Protein.

**Table S2. The relationship between CHG and the risk of MASLD or MetALD in different sensitivity analysis**

| Exposure | Model I(HR,95%CI) P | Model II(HR,95%CI) P-value | Model III(HR,95%CI) P-value |
| --- | --- | --- | --- |
| CHG (per 0.1-unit) | 1.064 (1.042, 1.088) <0.001 | 1.074 (1.050, 1.098) <0.001 | 1.086 (1.063, 1.110) <0.001 |
| CHG quartiles |  |  |  |
| Q1 | Ref | Ref | Ref |
| Q2 | 1.589 (1.249, 2.023) <0.001 | 1.630 (1.268, 2.094) 0.001 | 1.645 (1.280, 2.113) <0.001 |
| Q3 | 1.760 (1.384, 2.238) <0.001 | 1.714 (1.334, 2.203) <0.001 | 1.757 (1.368, 2.258) <0.001 |
| Q4 | 2.084 (1.638, 2.651) <0.00001 | 2.203 (1.718, 2.825) <0.001 | 2.379 (1.859, 3.043) <0.001 |
| P for trend | <0.001 | <0.001 | <0.001 |

Abbreviations: CHG: total cholesterol, high-density lipoprotein, and glucose; CI: confidence interval, Ref: reference; HR, Hazard ratios.

Model I was a sensitivity analysis in which **BMI was replaced with WC** in the model(n=6,274). Age, sex, WC, GGT, SBP, TG, ALT, HS-CRP, HbA1c, current smoking, physical activity, HTN, DM, DM-MED, DLP-MED, HTN-MED were adjusted.

Model II was a ensitivity analysis using **complete case data** (before multiple imputation; n=5,736). Age, sex, BMI, GGT, SBP, TG, ALT, HS-CRP, HbA1c, current smoking, physical activity, DM, DM-MED, DLP-MED, HTN-MED were adjusted.

Model III examined the association between **CHG and MetALD** ($n=7,927$). Age, sex, BMI, GGT, SBP, TG, ALT, HS-CRP, HbA1c, current smoking, physical activity, HTN, DM-MED, DLP-MED, and HTN-MED were adjusted.

**Table S3. Comparison of baseline characteristics between included and excluded participants**

| Characteristic | Included participants | Excluded participants | P-value |
| --- | --- | --- | --- |
| N | 6,274 | 17,391 |  |
| Age, years | 40.93 ± 9.35 | 41.04 ± 9.40 | 0.447 |
| SBP, mmHg | 114.36 ± 11.90 | 114.64 ± 12.02 | 0.118 |
| DBP, mmHg | 74.45 ± 7.79 | 74.64 ± 7.88 | 0.100 |
| BMI, kg/m² | 24.84 ± 3.26 | 24.88 ± 3.27 | 0.490 |
| WC, cm | 86.72 ± 10.71 | 86.77 ± 10.77 | 0.769 |
| TC, mg/dL | 193.88 ± 35.59 | 193.40 ± 35.41 | 0.355 |
| LDL-c, mg/dL | 120.06 ± 32.83 | 119.43 ± 32.77 | 0.188 |
| HDL-c, mg/dL | 52.06 ± 13.78 | 52.14 ± 13.93 | 0.674 |
| TG, mg/dL | 109.46 ± 59.72 | 109.76 ± 64.00 | 0.750 |
| FPG, mg/dL | 85.19 ± 9.20 | 85.46 ± 9.84 | 0.056 |
| Scr, mmol/L | 71.36 ± 14.94 | 71.72 ± 13.83 | 0.773 |
| AST, U/L | 27.37 ± 11.75 | 27.44 ± 13.19 | 0.714 |
| ALT, U/L | 31.00 (24.00-40.00) | 31.03 (24.01-40.78) | 0.259 |
| Hs-CRP, mg/L | 1.00 (0.50-2.30) | 1.00 (0.50-2.20) | 0.201 |
| GGT, U/L | 23.20 (16.91-32.78) | 23.00 (17.00-32.00) | 0.958 |
| DM, n% | 41 (0.65%) | 153 (0.88%) | 0.542 |
| HTN, n% | 416 (6.63%) | 1,236 (7.11%) | 0.353 |
| DLP, n% | 1,448 (23.08%) | 4,016 (23.09%) | 0.983 |
| HTN-MED, n% | 416 (6.63%) | 1,268 (7.29%) | 0.603 |
| DLP-MED, n% | 480 (7.65%) | 1,232 (7.08%) | 0.195 |
| DM-MED, n% | 85 (1.35%) | 282 (1.62%) | 0.621 |
| Current smoking, n% | 472 (7.52%) | 1,401 (8.07%) | 0.441 |
| Physical Activity, n% |  |  | 0.608 |
| Sedentary | 1,263 (20.13%) | 3,508 (20.17%) |  |
| Low | 2,225 (35.46%) | 6,298 (36.21%) |  |
| Moderate | 2,108 (33.60%) | 5,787 (33.28%) |  |
| High | 678 (10.81%) | 1,798 (10.34%) |  |
| Sex |  |  | 0.863 |
| Female | 2044 (32.58%) | 6,753 (38.83%) |  |
| Male | 4230 (67.42%) | 10,638 (61.17%) |  |

Continuous variables were summarized as mean (SD) or medians (quartile interval); categorical variables were displayed as percentage (%):Abbreviations: SBP, systolic blood pressure; HDL-c, High-Density Lipoprotein Cholesterol; AST, Aspartate Aminotransferase; LDL-c, Low-Density Lipoprotein Cholesterol; FPG, Fasting Plasma Glucose; BMI, Body Mass Index; TG, Triglycerides; WC, Waist Circumference; TC, Total Cholesterol; ALT, Alanine Aminotransferase; HS-CRP, High-Sensitivity C-Reactive Protein; DBP, diastolic blood pressure; Scr, Serum Creatinine; GGT, Gamma-Glutamyl Transferase; DM, Diabetes Mellitus; DLP, Dyslipidemia; HTN, Hypertension; HTN-MED, antihypertensive medication; DLP-MED, antihyperlipidemic medication; DM-MED, antidiabetic medication.

**Table S4. Comparison of characteristics of variables with missing values before and after imputation**

| Variables | Before imputation | After imputation | Number of missing values (%) | P-value |
| --- | --- | --- | --- | --- |
| WC (cm) | 87.31 ± 10.46 | 87.32 ± 10.33 | 35 (0.56%) | 0.988 |
| SBP (mmHg) | 114.60 ± 11.66 | 114.60 ± 11.65 | 27 (0.43%) | 0.988 |
| DBP (mmHg) | 74.52 ± 7.69 | 74.52 ± 7.69 | 27 (0.43%) | 0.975 |
| AST (U/L) | 27.75 ± 11.01 | 27.76 ± 10.99 | 123 (1.96%) | 0.966 |
| ALT (U/L) | 32.00 (25.00-41.00) | 32.01 (24.98-41.05) | 134 (2.14%) | 0.981 |
| Scr (µmol/L) | 70.79 ± 13.21 | 71.02 ± 13.44 | 139 (2.22%) | 0.875 |
| GGT (U/L) | 23.00 (17.00–33.00) | 23.02 (17.03–34.10) | 85 (1.35%) | 0.934 |
| Smoking (n, %) | 450 (7.54%) | 472 (7.52%) | 302 (4.81%) | 0.980 |
| DLP (n, %) | 1429 (23.22%) | 1466 (23.37%) | 121 (1.93%) | 0.852 |

Continuous variables were summarized as mean (SD) or medians (quartile interval); categorical variables were displayed as percentage (%). Abbreviations: WC, waist circumference; SBP, systolic blood pressure; DBP, diastolic blood pressure; AST, aspartate aminotransferase; ALT, alanine aminotransferase; Scr, serum creatinine; GGT, gamma-glutamyl transferase; DLP, dyslipidemia.

**Table S5. Stratified associations between CHG and the risk of MASLD by age, sex, BMI, AST, ALT, GGT, SBP, DBP, smoking status, DM-MED, DLP, and Physical Activity**

| Characteristics | participants | HR (95%CI) *P* value | *P* for interaction |
| --- | --- | --- | --- |
| Age(years) |  |  | 0.2686 |
| <30 | 404 | 1.093 (0.975, 1.226) 0.1282 |  |
| 30-40 | 2527 | 1.076 (1.040, 1.112) <0.0001 |  |
| 40-50 | 2292 | 1.041 (1.008, 1.075) 0.0147 |  |
| 50-60 | 902 | 1.020 (0.974, 1.067) 0.4021 |  |
| ≥60 | 149 | 1.037 (0.936, 1.148) 0.4919 |  |
| Sex |  |  | 0.9646 |
| Female | 2044 | 1.049 (0.990, 1.111) 0.1041 |  |
| Male | 4230 | 1.047 (1.021, 1.075) 0.0004 |  |
| Smoking |  |  | 0.1662 |
| No | 5802 | 1.083 (1.059, 1.109) <0.0001 |  |
| Yes | 472 | 1.040 (0.983, 1.100) 0.1769 |  |
| BMI categories |  |  | 0.1236 |
| <18.5 kg/m^2^ | 582 | 1.251 (1.033, 1.516) 0.0221 |  |
| 18.5-24 kg/m^2^ | 1919 | 1.077 (1.008, 1.151) 0.0274 |  |
| 24-28 kg/m^2^ | 2900 | 1.044 (1.016, 1.074) 0.0023 |  |
| ≥28.0kg/m^2^ | 873 | 1.059 (1.011, 1.108) 0.0143 |  |
| SBP |  |  | 0.1685 |
| <140 mmHg | 5992 | 1.060 (1.034, 1.086) <0.0001 |  |
| ≥140mmHg | 282 | 1.085 (1.013, 1.161) 0.0190 |  |
| DBP |  |  | 0.2458 |
| <90 mmHg | 5957 | 1.060 (1.034, 1.086) <0.0001 |  |
| ≥90mmHg | 317 | 1.005 (0.922, 1.096) 0.9037 |  |
| AST |  |  | 0.7217 |
| <40u/L | 5856 | 1.083 (1.057, 1.109) <0.0001 |  |
| ≥40u/L | 418 | 1.071 (1.010, 1.136) 0.0226 |  |
| ALT |  |  | 0.1456 |
| <40u/L | 4497 | 1.090 (1.056, 1.126) <0.001 |  |
| ≥40u/L | 1777 | 1.081 (1.052, 1.111) <0.001 |  |
| GGT |  |  | 0.4492 |
| High | 5799 | 1.083 (1.058, 1.110) <0.0001 |  |
| Low | 475 | 1.059 (0.999, 1.122) 0.0555 |  |
| DM-MED |  |  | 0.5520 |
| No | 6233 | 1.053 (1.028, 1.079) <0.0001 |  |
| Yes | 41 | 1.111 (0.934, 1.322) 0.2344 |  |
| DLP |  |  | 0.0893 |
| No | 4808 | 1.067 (1.038, 1.097) <0.0001 |  |
| Yes | 1466 | 1.031 (0.995, 1.068) 0.0957 |  |
| Physical Activity |  |  | 0.7042 |
| Sedentary | 1154 | 1.035 (0.993, 1.080) 0.1046 |  |
| Low | 2382 | 1.054 (1.019, 1.089) 0.0020 |  |
| Moderate | 2120 | 1.067 (1.031, 1.105) 0.0003 |  |
| High | 618 | 1.060 (0.989, 1.136) 0.1010 |  |

Note 1: Above model adjusted for age, sex, BMI, GGT, SBP, TG, ALT, HS-CRP, HbA1c, current smoking, physical activity, HTN, DM, DM-MED, DLP-MED, HTN-MED.

Note 2: In each case, the model is not adjusted for the stratification variable.

Abbreviations: HR, Hazard ratios; CI: confidence, Ref: reference; SBP, systolic blood pressure; DBP, diastolic blood pressure; DM-MED, antidiabetic medication; DLP, Dyslipidemia. AST, Aspartate Aminotransferase; BMI, Body Mass Index; GGT, Gamma-Glutamyl Transferase; ALT,Alanine Aminotransferase.

**Table S6. Results of the two-piecewise Cox proportional hazards regression model in participants not using DM medications or lipid-lowering medications**

| Outcome: Incident MASLD | Model I, HR (95%CI) p-value | Model II, HR (95%CI) p-value |
| --- | --- | --- |
| Inflection points of CHG | 5.42 | 5.42 |
| < 5.42(per 0.1-unit) | 1.097 (1.061, 1.135) <0.001 | 1.088 (1.054, 1.123) <0.001 |
| ≥5.42(per 0.1-unit) | 0.982 (0.923, 1.044) 0.556 | 0.965 (0.907, 1.026) 0.259 |
| P for log-likelihood ratio test | 0.003 | 0.001 |

Model I was a sensitivity analysis after excluding participants using **DLP-Med** (N =5,794). The model was adjusted for age, sex, BMI, GGT, SBP, TG, ALT, HS-CRP, HbA1c, current smoking, physical activity, HTN, DM, DM-MED, HTN-MED.

Model II was a sensitivity analysis after excluding participants using **DM-Med** (N =6,189). The model was adjusted for age, sex, BMI, GGT, SBP, TG, ALT, HS-CRP, HbA1c, current smoking, physical activity, HTN, DM, DLP-MED, HTN-MED.

**Table S7. Time-dependent ROC curves of baseline CHG for predicting incident MASLD within 2.0, 3.0, 4.0, and 5.0 years.**

| Follow-up(years) | N | events | AUC (95%CI) | Best threshold | Specificity | Sensitivity | Youden index |
| --- | --- | --- | --- | --- | --- | --- | --- |
| 2.0 | 6274 | 657 | 0.656 (0.635–0.677) | 5.12 | 0.686 | 0.555 | 0.242 |
| 3.0 | 6274 | 912 | 0.666 (0.647–0.683) | 5.17 | 0.638 | 0.617 | 0.255 |
| 4.0 | 6274 | 994 | 0.669 (0.651–0.687) | 5.17 | 0.635 | 0.620 | 0.255 |
| 5.0 | 6274 | 1104 | 0.678 (0.662–0.695) | 5.17 | 0.642 | 0.627 | 0.269 |

Abbreviations: AUC: area under the curve; CI: Confidence interval; CHG: total cholesterol, high-density lipoprotein, and glucose.

**Table S8. Comparison of Baseline Characteristics of Participants with CHG<5.42 and CHG≥5.42**

| CHG | <5.42 | ≥5.42 | P-value |
| --- | --- | --- | --- |
| N | 5141 | 1133 |  |
| Age, years | 40.66 ± 8.62 | 42.10 ± 8.14 | <0.001 |
| SBP, mmHg | 113.52 ± 11.36 | 119.48 ± 11.71 | <0.001 |
| DBP, mmHg | 73.86 ± 7.54 | 77.53 ± 7.64 | <0.001 |
| BMI, kg/m² | 24.54 ± 3.05 | 26.37 ± 2.86 | <0.001 |
| WC, cm | 85.92 ± 10.39 | 93.60 ± 8.25 | <0.001 |
| TC, mg/dL | 187.65 ± 31.83 | 225.08 ± 35.80 | <0.001 |
| LDL-c, mg/dL | 113.70 ± 28.74 | 152.83 ± 33.97 | <0.001 |
| HDL-c, mg/dL | 54.52 ± 12.97 | 38.16 ± 6.72 | <0.001 |
| TG, mg/dL | 97.37 ± 41.49 | 172.93 ± 125.75 | <0.001 |
| HbA1c, % | 4.57 ± 0.27 | 4.82 ± 0.49 | <0.001 |
| FPG, mg/dL | 84.24 ± 7.78 | 91.44 ± 14.13 | <0.001 |
| AST, U/L | 27.44 ± 11.50 | 29.23 ± 8.15 | <0.001 |
| ALT, U/L | 33.81 ± 15.54 | 41.15 ± 17.82 | <0.001 |
| HS-CRP, mg/L | 2.15 ± 4.95 | 2.33 ± 6.18 | <0.001 |
| GGT, U/L | 27.68 ± 21.42 | 38.44 ± 28.16 | <0.001 |
| DM, n% | 22 (0.43%) | 19 (1.68%) | <0.001 |
| HTN, n% | 310 (6.03%) | 102 (9.00%) | <0.001 |
| DLP, n% | 1030 (20.04%) | 436 (38.48%) | <0.001 |
| HTN-MED, n% | 321 (6.24%) | 95 (8.38%) | 0.009 |
| DLP-MED, n% | 410 (7.98%) | 70 (6.18%) | 0.039 |
| DM-MED, n% | 62 (1.21%) | 23 (2.03%) | 0.030 |
| Current smoking, n% | 353 (6.87%) | 119 (10.50%) | <0.001 |
| Physical Activity, n% |  |  | <0.001 |
| **Sedentary** | 909 (17.68%) | 245 (21.62%) |  |
| **Low** | 1918 (37.31%) | 464 (40.95%) |  |
| Moderate | 1767 (34.37%) | 353 (31.16%) |  |
| High | 547 (10.64%) | 71 (6.27%) |  |
| Sex |  |  | <0.001 |
| Female | 1977 (38.46%) | 67 (5.91%) |  |
| Male | 3164 (61.54%) | 1066 (94.09%) |  |

Continuous variables were summarized as mean (SD) or medians (quartile interval); categorical variables were displayed as percentage (%):Abbreviations: SBP, systolic blood pressure; DBP, diastolic blood pressure; WT, Weight; HT, Height; BMI, Body Mass Index; WC, Waist Circumference; TC, Total Cholesterol; LDL-c, Low-Density Lipoprotein Cholesterol; HDL-c, High-Density Lipoprotein Cholesterol; TG, Triglycerides; HbA1c, Hemoglobin A1c; FPG, Fasting Plasma Glucose; AST, Aspartate Aminotransferase; ALT, Alanine Aminotransferase; Scr, Serum Creatinine; HS-CRP, High-Sensitivity C-Reactive Protein; GGT, Gamma-Glutamyl Transferase; DM, Diabetes Mellitus; DLP, Dyslipidemia; HTN, Hypertension; HTN-MED, antihypertensive medication; DLP-MED, antihyperlipidemic medication; DM-MED, antidiabetic medication; CHG，total cholesterol, high-density lipoprotein, and glucose.


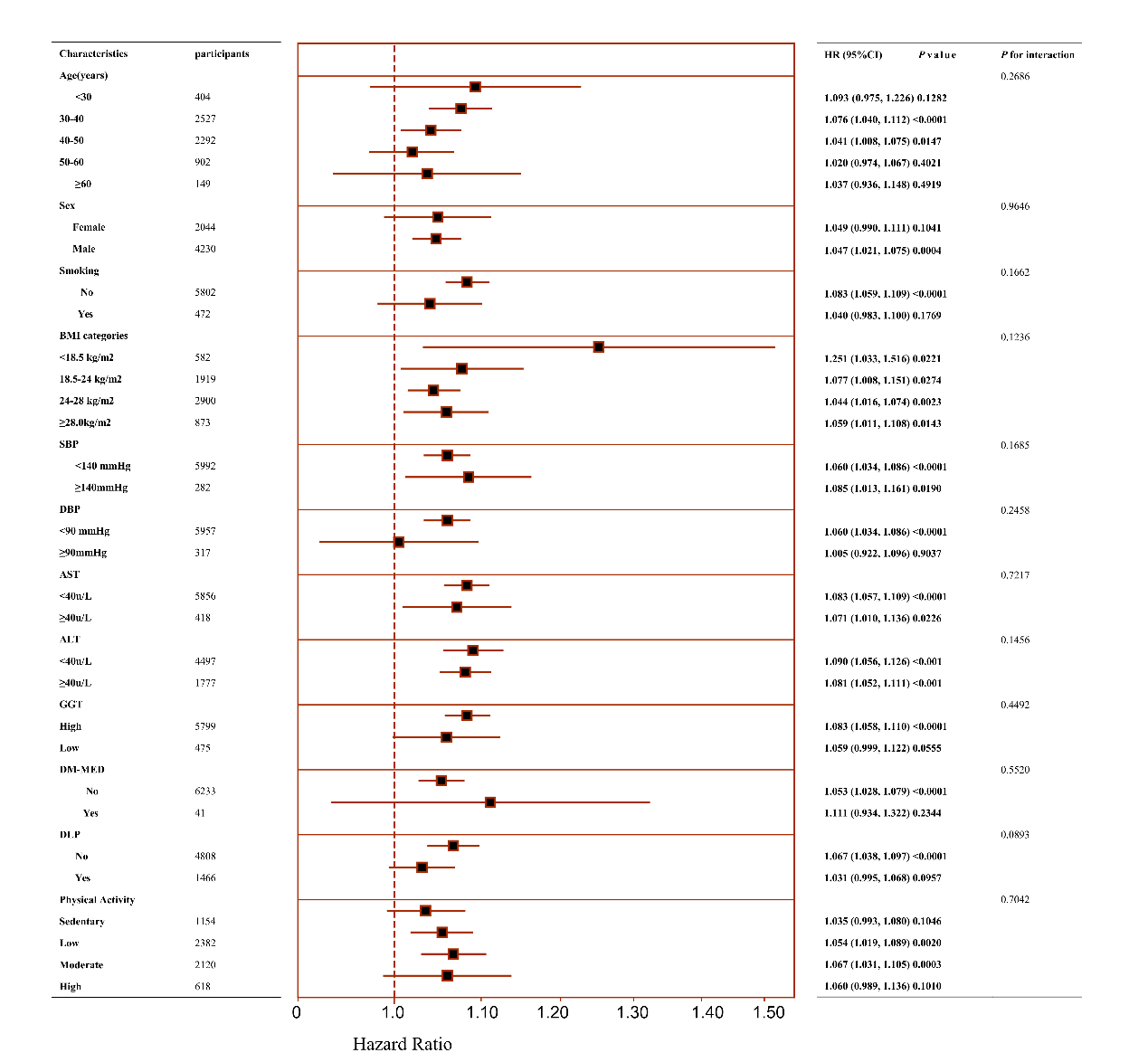


**Figure S1: Forest plot of the association between CHG and MASLD risk stratified by age, sex, BMI, AST, ALT, GGT, SBP, DBP, smoking status, DM-MED, DLP, and physical activity**


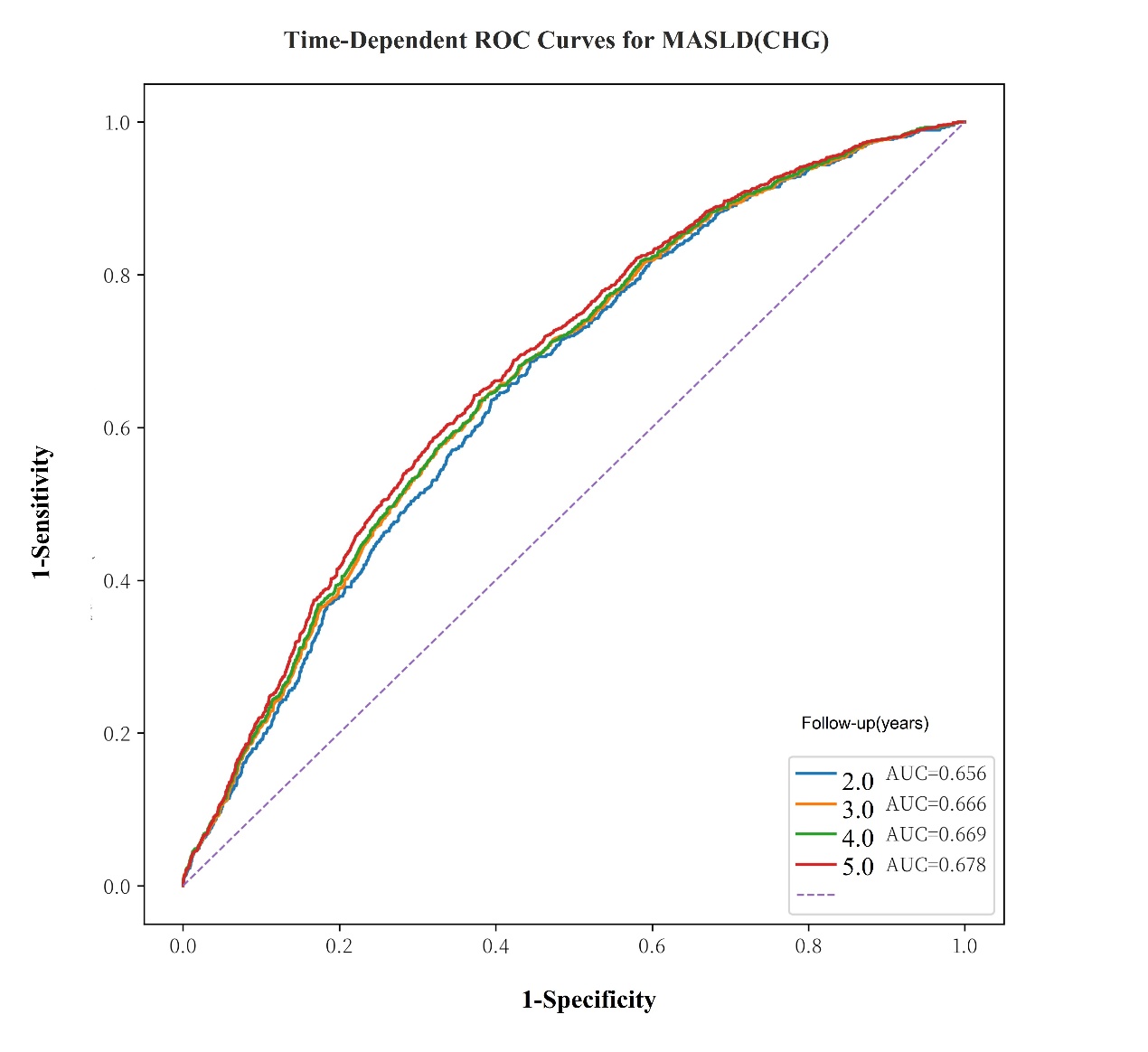


**Figure S2: Time-dependent ROC curves for CHG prediction of MASLD**
